# Casualty Surgeon

**Published:** 1962

**Authors:** H. K. Bourns

**Affiliations:** Consultant Surgeon in Charge of Casualty Department, United Bristol Hospitals


### CASUALTY SURGEON

BY

H. K. BOURNS, B.A., M.B., F.R.C.S.

*Consultant Surgeon in Charge of Casualty Department,*
*United Bristol Hospitals*

The *Oxford Dictionary* gives the meaning of the noun Casualty as an accident, mishap or disaster. The adjective is casual, meaning accidental, irregular, undesigned, unemotional, careless. I had chosen the title for this lecture before I read the dictionary and because the term "Casualty Surgeon" has been very close to me for many years. Now I know that when your Secretary asked me to speak to this learned Society, it was a mishap, and when I accepted it, it was a disaster. Perhaps before the end of the lecture you will feel that the whole proceedings have been both irregular and undesigned.

*Casualty Surgeon*

When one looks for the reason for a Casualty Surgeon, one knows that Hospital Management Committees and Boards, who have considered Casualty work, have made a senior appointment in order that they may be covered from criticism and may have someone on whom they can shed responsibility. A Casualty Department is a heavy commitment for any hospital and it is a recurring source of trouble for the administrators. The United Bristol Hospitals, in its wisdom, did not appoint a Casualty Surgeon but a Surgeon in Charge of the Casualty Department. There is a big distinction here. To appoint a Casualty Surgeon implies that there is a particular branch of surgery which is performed in Casualty and which cannot be carried out anywhere else. In other words, there is a Speciality called Casualty Surgery. I think that this is the "Casualty Dilemma"[1] which is attracting the attention of many to-day. Is the Casualty Department a place for special treatment and a situation in which a medical man can specialize in a useful capacity? All know the definition of a specialist as a man who knows more and more about less and less until eventually he knows everything about nothing. Is this the sort of position to be attained by a Casualty Surgeon, or is there some branch of surgery in which a Surgeon in charge of Casualty can find a life interest?

Before we can answer this question, I feel that we must examine the present-day departments. Since the inception of the National Health Service, in which everyone has a doctor and every region is said to have a hospital service organized in clinical and other areas, one might have expected the need for Casualty Departments to have passed away as something only needed in the bad old days. In fact, Casualty Departments are as busy as ever, but I do not propose to examine the reasons for this state of affairs.

Many have analysed attendances[2]—we have done so in Bristol. Approximately 50 per cent of our cases are traumatic in origin, 10 per cent are infections of various descriptions, 5 per cent abdominal emergencies, 14 per cent are wounds and about 5 per cent are cysts, skin lesions, ganglions and circumcisions. In an ordinary week, the following cases were dealt with in the Casualty Department of the Bristol Royal Infirmary:

| | | | |
|---|---|---|---|
| Injuries .. .. | 428 | Abdominal pain .. | 39 |
| Fractures .. .. | 20 | Dental .. .. | 32 |
| Lacerations .. | 59 | Splinters .. .. | 11 |
| Septic hands .. | 12 | Swallowed tablets .. | 12 |
| Other sepsis .. | 20 | Collapse .. .. | 24 |
| Dog bites .. .. | 10 | TOTAL | 756 |

The kind of accidents dealt with is shown by the following analysis of accident cases admitted during six months:

| | | | |
|---|---|---|---|
| Fractures .. .. | 336 | Head injuries .. | 239 |
| Sprains and strains | 16 | Nerve injuries .. | 4 |
| Contusions .. | 32 | Internal injuries .. | 15 |
| Open wounds .. | 87 | Superficial injuries | 39 |
| Dislocations .. | 25 | Poisoning .. .. | 59 |
| Traumatic amputations | 3 | Foreign bodies .. | 3 |
| Burns .. .. | 19 | Arterial injury .. | 1 |

What is the role of a Casualty Surgeon in all this? It is laid down in the Hospital Planning Notes for Provision and Design of Casualty and Accident Departments sections 23–(1).[3] "The Department will be in charge of a Consultant Surgeon who, working with appropriate staff, will provide immediately consultant cover through 24 hours." If one Surgeon were to do this he could neither eat, sleep nor drink. Surgeons are expendable, but none could stand this pace for very long and so the supply would run out.

However, in these notes we come to the title "Casualty and Accident Departments", and this is a distinction that will make a rational and practical solution possible. A large number of casualty cases are accidents or emergencies and it is for these that we must develop a service. It is these who demand immediate attention if more are to be saved and progress is to be made. In fact, a casualty doctor must know something about everything.

### *The Function of Casualty Departments*

Casualty Departments are not places to be developed as palaces or empires of a particular surgeon or group of surgeons. The space must be kept clear for the new cases and must not be occupied by non-urgent cases and the staff must not be occupied in performing surgery in the department on patients who could be easily accommodated elsewhere. This is the ideal but it can only be acheived when provision is made for some surgery for all who work in the department and when the department is an integral part of the hospital to which it belongs. Lamont[1] recommends an integrated self-contained Casualty Service, preferably on a national basis, in which the acolytes can be trained in the subtleties of the work. My preference is for a National Casualty Policy rather than a self-contained service. But he goes on: "What matters most, however, is that each casualty unit should have at its head a Consultant of high attainment . . . his primary function, apart from any special operative skills he may possess, is to be a father figure to the young men who will make their career in the Casualty Service."

In my opinion, this is the wrong approach. We must advance to Accident and Emergency Services attached to general hospitals and in charge of surgeons who, in addition to their responsibilities to the department, would practise surgery. It does not matter what branch of surgery a man in charge of Casualty takes up so long as he is interested in the department and some branch of the work in it. A Casualty Department requires a Consultant in charge if only to administer and to advise on equipment and policy. In this sphere, as in every other, advances are being made and constant attention is needed to avoid waste and the installation of untried remedies. We must always adopt a questioning attitude to all remedies and practices, but travellers from drug firms seem to regard Casualty as a happy hunting ground. They can easily slip in through the ever open door and accost either Sister or doctor. They paint glowing pictures of their newest product and dispense freely, so that the Casualty Officer, who is flattered by this attention, can readily apply the new treatment. So often he leaves before he has had time to assess its value, and the new Casualty Officer is fair game for the same gambit.

*The Work of a Casualty Department*

Surgery coming into the Department falls into five main groups:

1. Traumatic soft tissue surgery.
2. The treatment of sepsis.
3. Minor surgery.
4. Special types of trauma—e.g. burns and scalds, fractures.
5. General surgical emergencies.

*Trauma* divides itself into major and minor. I will not spend too much time on major except to remind you that restoration of the patient's blood volume is a basic principle in nearly all cases. We often make impossible demands on the Blood Transfusion Department, but the late Ruscoe Clarke[4,5] in the Birmingham Accident Department emphasized the need for blood replacement and maintenance of blood pressure no matter what the injury seemed to represent. This, of course, means accurate diagnosis and assessment, and no doubt many of you will realize that it requires a much more experienced person to decide what to do than to do it. A recent case illustrates this point. A patient was brought to the Casualty Department with the history that a van had backed slowly into her as she stepped off the pavement. She was knocked down and her left lower limb was injured. There were no bones broken and the skin was avulsed from the leg below the knee. She was deeply shocked and a transfusion was commenced and given slowly. Rest with the foot of the bed elevated and morphia produced a temporary rise of blood pressure, but then this began to fall again. In the operating theatre the cause for this was found when the full extent of the injury was seen. Avulsion of all skin and subcutaneous tissue on the left lower limb extending up to and including the buttock was found. A three hour operation and 14 pints of transfused blood and plasma were necessary to graft the whole area and to restore the circulation. A good result was obtained but the operation would not have been possible without the large transfusion, and the healing of the patient would have been retarded without the rapid restoration of her blood volume. Such a case makes a sudden demand which will be beyond the scope of any hospital that does not make full provision for accident cases. This case also illustrated a problem of staffing. While this woman was being treated other patients were arriving. It took four medical persons approximately three hours to deal with this woman, and there must be sufficient staff available to treat such a case and to keep the Department functioning at the same time.

Delayed primary treatment is a very useful manoeuvre and has been made possible by the use of antibiotics, chymotrypsin,[6] and hospitalization for traumatic cases. Skin grafting must be used freely in order to obtain early healing.

A sixteen-year-old who injured his left hand in a concrete mixer had a lesion in which it was impossible to be certain of the full extent of the damage when he was first seen. The skin from the forearm downwards was disturbed and bruised. It might have been so damaged as to die, but after 48 hours it was obvious that nearly all the skin was viable, and so a skin graft to the defect was performed. Healing was obtained in approximately six weeks and the hand was restored to full function after approximately nine months. If immediate operation had been performed an unnecessarily large operation would probably have been performed; by waiting for 48 hours delayed primary or definitive treatment was carried out when all viable structures were evident, and only those that were dead had to be removed. Chymotrypsin[6] is useful in surgery to reduce venous thrombosis and to prevent organization of the tissue exudate in bruised tissues. It must, however, be given within four hours of the injury and must be given in doses of 5,000 units intramuscularly 6-hourly for two to three days. If it is not given during the first twenty-four hours, it is probably of little avail.

*Hand Injuries*

Hand injuries are a particular problem, and I am sure that there are now established rules and principles which we must try to follow. Early skin cover is vital. Preservation of the living tissue and time for the temporarily stunned to recover is part of this problem. Hand injuries are divided into clean and dirty. The clean are those in which the tissues are cut cleanly no matter how badly. The dirty are those with crushing, bursting, contusion and damage to tissue which may or may not live and heal. In the clean, immediate neat and tidy surgery may be possible. In the second type either dressing only or general débridement is the immediate necessity, with delayed primary or secondary procedures carried out later, when recovery is evident. Skin closure to minimize scarring is the first essential.

The injured finger represents a separate problem. The treatment must take into consideration age, sex, occupation, the time that a particular treatment will take, the finger involved, and the hand. Treatment may be altered if bone or tendon is injured, or if a sensory nerve is also injured. If amputation is a possibility this must be given very early and careful consideration. Psychologically the patient will often accept this as inevitable at the time of the injury, but refuse it later. Against this is the fact that a state less than full function may be all that a patient requires when he returns to his pre-accident work.

In partial amputations of finger, the grafting of any available skin 48 hours after the injury may be necessary if healing is to be obtained in a reasonable time. However, a recent article in the *British Medical Journal*[7] endorsed what we have felt in Bristol—that is that an amputation stump healed by flaps is the best type of finger-end, and split skin graft comes next. Indiscriminate burying of an odd finger in the abdominal wall, popular some years ago, is to be condemned as time consuming and wrong.

If, in a working man, skin closure can be obtained by shortening a phalanx by a few millimetres, this should be carried out. Healing is rapid and disability minimal. If this brings the level of bone section to a few millimetres from a joint, then disarticulation at the joint is the best procedure because a cap of bone distal to an interphalangeal joint is useless. It is immobile, sticks out, and stiffens the finger. In a joint, preserve the articular cartilage on the end; it does not interfere with healing, and it reduces the chance of sepsis in the bone because the cancellous bone in the shaft of the phalanx is not opened to the wound and the potential infection. If a finger lacks sensation then it is a menace to a man working with machines; amputation may be the wisest course. Preservation at all costs of portions of fingers may not be in the best interest of the man or his mates. Compound fractures with skin and tendon loss will always lead to stiffness no matter what treatment is given, so again amputation may have to be considered.

Cosmetic results must be given full consideration, but the female and the highly skilled male are those who may be more interested in appearance than function. Recently a mannequin mishandled sepsis in the proximal interphalangeal joint of the ring finger of the right hand. The end of the finger sloughed. She demanded a stump which would be long enough to take a prosthesis in order that she might be able to display gloves and be able to demonstrate with a hand which had four fingers. She felt that the complete absence of one finger might be a disadvantage in her occupation. Amputation through the neck of the metacarpal is a satisfactory cosmetic result in most circumstances, and amputation through the metacarpo-phalangeal joint, though more noticeable, ensures more power because it preserves the palm.

*Tendon Injuries*

In flexor tendon injuries it is essential to obtain primary healing of the skin and then carry out secondary sutures if the situation is suitable and demands it. The fourth and fifth fingers are considered separately because if there is tendon damage, one must try to assess the extent of the disability in relation to the age and work of the patient in order to decide on the operation to be performed. Rupture of a *profundus* tendon means loss of flexion of the distal joint only. Often stability is as important as movement to a patient, and so arthrodesis of the distal joint in slight flexion is very useful because it gives a powerful finger. One can suture the *profundus* end to end anywhere other than in the area between the metacarpo-phalangeal joint and the proximal interphalangeal joint. An intact *sublimis* must never be sacrificed for a *profundus* repair so that if the *profundus* is divided in "no man's land" a tendon graft is the only possibility. In the young, an attempt to restore normal function is justified no matter which finger or fingers are involved because one does not know what occuaption they may eventually wish to follow and because they have time to heal and regain function.

In the thumb a *flexor pollicis longus* tendon should always be either sutured or grafted. The result will always be good even if stiff, because a stable thumb in slight flexion as a pincer or lever may be all that is required. In many such cases the patient is more satisfied with the result than the surgeon.

A ruptured extensor of a distal joint leads to a mallet finger deformity. Surgery is difficult and splinting will not produce a first class result. If there is a piece of bone avulsed from the distal phalanx with the tendon, replacement by open operation and fixation with wire may give a good result. If there is avulsion of the tendon from the phalanx, protection while the part is painful after trauma is the better immediate treatment. If the patient is not satisfied later, something may be done by open operation but only after the patient has been made to realize that the treatment does not give much more than a 50 per cent chance of success. Delay provides scar tissue which can be used in the repair. At the proximal interphalangeal joint surgery is useful. Either suture or re-routing the lateral slips over the dorsum of the joint is satisfactory, and although it may not give a perfect result it will make the finger more useful.

Fractured phalanges and metacarpals should be fixed with intramedullary pins if unstable. This eliminates the need for plaster or other splints and allows early mobilization if this is desirable. One must however relate all treatment to function; and this is related not only to anatomy, but also to the job. If a finger or hand works, leave it alone.

*Sepsis*

Carbonet,[8] developed at the Bristol Royal Infirmary, is one of the greatest recent advances in dressing septic wounds or superficial abscesses and ulcers. It prevents the soggy oedematous wounds and maceration of the skin which used to be seen in the days of repeated dressing with gauze impregnated with soft paraffin. Most of the dressing gauzes now have "water miscible" bases, and the healing wound is no longer delayed by greasy dressings.

Studies of staphylococci in the Casualty Department proceed all the time, and the results of a recent survey are published in the *Journal of Clinical Pathology*.[9] It has been shown that the phage type of staphylococcus is important because on this the course of an infection depends. In our investigation we demonstrated the interesting fact that localization takes almost the same time no matter what the organism is. We also showed that sloughing is related more to the organism than to the treatment or blood supply of the part. Penicillin is useful and no one has proved that it is not. Anderson[10] stated in 1958 that "apparently routine chemotherapy was not of major importance provided special attention was paid to careful clinical examination, accurate diagnosis and precise surgery". Everyone would agree with the points about examination and treatment, but antibiotics cannot be condemned in hand surgery and sepsis even if they only play a secondary part. In fact, we have all fallen into the error of expecting too much of penicillin. In some of our minds the idea of either penicillin or surgery has developed—"If penicillin fails then try surgery". Antibiotics and surgical treatment should be used simultaneously and should not be regarded as alternative methods.

What is the place of penicillin in the treatment of hand infections? This is the problem which is being investigated at present. Is oral administration as satisfactory as intramuscular? We seem to have come down to a basic treatment time below which it may be impossible to go, once pus has formed. Localization, incision and healing seems to take about 8 days and no one seems to be able to better this. Elucidation of the progress of these infections is important, so that we may have firm indications for the use of antibiotics. The results of a recent trial of about 1,000 cases will soon be published and a plan of treatment will be laid down. An initial injection of penicillin followed by oral administration would seem to be the best course to follow.

*Minor Surgery*

Minor surgery is impossible to define. Is it minor because the incisions are small and the damage easily repaired or paid for? Minor surgical cases require some skill and experience, and their conduct is very important. In the B.R.I. Casualty Department we operate on an average of about 150 cases per month; some two-thirds of these operations are performed under local anaesthesia. It is unfortunate if a patient with a sebaceous cyst takes up a precious place in an out-patient clinic where a surgeon is holding consultations. Yet it is certain that a consultant or senior hospital officer should be responsible for this branch of surgery. Not all cysts are what they seem, and even a consultant can make a mistake. Professor Hewer[11] published the results of histological examinations of specimens received by his department from Casualty during a period of 12 months. Lists such as this are normally boring and only interesting to the person who produces them, but the observation and treatment of such a large group of superficial lesions is a very useful exercise and it is obvious that interest in minor surgery is rewarding. For instance the diagnosis and treatment of "mucoid cysts" of the finger results in almost 100 per cent cure now that we recognize excision without closure as the only satisfactory method. All else has failed, and radiotherapy, as recommended in 1960[12] is not justifiable.

Ganglions on the back of the wrist are common and well known. The smaller and less common one on the anterior aspect of the proximal phalanx of the finger seems to be less well known. It is often regarded by the Casualty Officers as bony, but a bony ossicle in front of the flexor sheath is unknown, and the fact that normal movement of the tendons is present makes the diagnosis easier. Incidentally, the treatment of ganglions is surgical excision; bursting of the capsule is a very second best. A benign calcified epithelioma is a very peculiar little lesion. It occurs most frequently in young people. I have seen a number in children in whom a small hard calcified lesion has developed on the scalp. It is noteworthy that neuro-fibromata and neuro-fibrosarcomata are almost as common as melanomata. They are almost as dangerous. They may undergo malignant change, and close co-operation with the pathologist is very important in dealing with these cases.

So-called "phimosis" used to be the preserve of the House Officers and the out-patient Sister in the past, and this state of affairs may continue in some smaller hospitals. The anti-circumcision drive was largely prompted by the blind approach to the indications for, and to the performance of, the operation. Phimosis does exist, but often does not require circumcision. Phimosis due to fibrosis of the prepuce must be relieved by surgery. It may occur as a result of repeated and vigorous stretching by a parent or district nurse and it may develop in later life because of atrophy of tissues in diabetes and other conditions. Recurrent balanitis may also be a cause but this condition is rare. Irritation and soreness occur but are caused by the retention of smegma and débris in an unseparated prepuce. The débris occurring on the corona of the glans leads to itchiness, the child responds by rubbing, and this leads to soreness. Complete separation under an anaesthetic is the remedy. Cleaning is then easy and continues automatically in the bath. This usually solves the problem. It should be remembered that circumcision is not the only operation; a dorsal slit is a very useful procedure and gives a satisfactory result. When performing a circumcision, it must be remembered that some prepuce should be left to protect the corona of the glans. After operation, plastic pants save the bed and clothes of parents and relatives, but they lead to soreness of the prepuce and excoriation if worn for 24 hours a day. They should not be left on all night, and a mackintosh draw-sheet should be used.

For minor surgery close co-operation with the pathology department is very necessary and although one should not rush to large operations, excision-biopsy is a very useful procedure. To take snips may not give the pathologist a chance, and if the lesion is malignant it may, in some cases, cause spread outside the future operation area. One must not be afraid of adequate excision. Where malignancy is concerned life is often at stake, and so the right operation must be performed even though the patient is sometimes surprised by its extent.

Tuberculous lymphadenitis should not be forgotten. It is not so common nowadays, but with sterile abscesses this possibility should not be overlooked. The pus is sterile on culture, and special culture or inoculation gives a delayed answer. Immediate proof can often be given on microscopic examination of the wall of an abscess or cavity. Speed is important because if chemotherapy is to be of any value, it must be used in conjunction with surgery. It is generally accepted that tuberculous lymphadenitis is unlikely to be cured by chemotherapy alone. The patient may overcome the trouble without any treatment, but if excision is practised, that is the time to use specific chemotherapy. However, diagnosis is the ever-present problem. With antibiotics many pyogenic abscesses are sterile, so biopsy of the wall is essential. The sites are interesting—location in the axilla or groin, is almost as common as in the neck glands. In ten years we have seen only two primary skin lesions so that the source of the glandular infections is difficult to surmise.

*Other Emergencies*

Medical and mixed medical and surgical emergencies form a group of problem cases. Cardiac arrest in coronary ischaemia is a problem because one must be prepared to carry out immediate resuscitatory measures, using artificial mouth to mouth or endotracheal respiration and external cardiac massage. A Brooks or similar mouth piece should in the pocket of every doctor since this mouth to mouth method of respiration is now widely known and very beneficial. The rapid diagnosis and treatment of medical emergencies is a serious problem and one wonders why someone has not suggested the appointment of a Casualty Physician. Cases have occurred of coronary artery occlusion in young men in their thirties whose hearts stopped whilst in the Casualty Department, and who were resuscitated with cardiac massage and positive pressure respiration. The surgery of coronary artery disease is in its infancy but it can only advance if the body can be preserved alive until the patient is fully assessed and transported to an efficient cardiac unit with all the necessary equipment.

Cases of poisoning demand expert attention too and the setting up of Regional advisory centres where information can be collected and so arranged that it can be readily available to all concerned, is a vital necessity. Treatment must be related to the cause of the toxic state, but the number of noxious agents has increased so rapidly recently that no one can hope to know their form or presence in all the various proprietary and other preparations to be found in the home and countryside. Regional Centres will provide an up-to-date information service, and can collect data on the properties of any agent so that the dosage which is likely to cause damage will be known and the most successful treatment will be discovered as early as possible. A twenty-four-hour telephone service is conceived and this should soon be available in every region.

### THE FUTURE

In the future minor surgery must be separated from the accident and emergency centre. I am sure that we must develop an out-patient clinic to which patients can be referred for treatment without long delay for appointments. These clinics would be the responsibility of surgeons who are interested or who had been appointed with the supervision of minor surgical treatment as part of their contract. We must not allow these cases to drift unsupervised, but an accident centre is going to be too busy for them and the patients are out of place in such a department. In the future we hope to separate these problems, but we must make arrangements for minor surgery because without health centres or other means of dealing with these patients outside the hospitals, the out-patients clinics might be suddenly invaded by a large group of people. Is this a point where G.P.s and hospital staff could meet? Is this surgery beneath the dignity of a G.P.? To those who are interested this might be a point of contact, because of course it could easily be arranged on a sessional basis, and time for discussion and consultation would surely follow.

The British Orthopaedic Association[14] reports that accident cases should be seen by "a clinician experienced in two respects: (1) he must be able to distinguish between the minor injury and the injury that is actually or potentially serious. (2) This clinician must also be competent to deal efficiently with minor injuries—the small superficial burn, the Colles's fracture, the sprained ankle." The report says "a surgeon resident in a small town or a general practitioner is the right man for this work, provided that his training in accident surgery is adequate." That paragraph would apply equally to minor surgery. This is not a speciality, and yet surgery of this sort must be performed adequately and supervised by a full member of the hospital staff. If any hospital is to take responsibility for such cases, then they must be fully integrated into the out-patient services rendered by its consultants. At present, many people lack confidence in the safety of any surgery, however trivial, performed outside a hospital, and the remedy for the separation of the general practitioner and patient may be for both to use the hospital facilities.

The accident service of this country requires re-organizing. Small centres can give resuscitation to major cases and treatment to minor, but there must be a proper service organized on a geographical and regional basis. These centres are for accidents and emergencies and must be staffed and geared for this. They must be attached to larger hospitals and surgeons and physicians on the staff must take part in the service on a rota basis. Casualty Surgeons as such must disappear, if they ever existed, and although any one centre must be in charge of a consultant, this will be for administration and ease of direction only, and not because he and he alone is treating trauma in that centre. As far as treatment is concerned teams of surgeons, physicians, and anaesthetists are desirable, all working together and knowing their own parts without rehearsal or confusion. In this way lives will be saved and experience accumulated in a field where identical cases do not recur very often.

I have tried to demonstrate the interest that lies in many of the facets of "casualty" as we know it at present. There is interest for any member of the medical profession who is prepared to see it. My old Professor of Surgery was never tired of quoting rather irreverently the passage from Jeremiah about "eyes have they and they see not, ears have they and they hear not" and he used to add his own invention "hands have they and they feel not". This can be applied to Casualty—the "shop window of the Health Service".

### REFERENCES

1. Lamont, D. (1961). *Lancet*, **2**, 1190.
2. Mestitz, P. (1957). *Brit. Med. J.*, **2**, 1108.
3. Accident Services of Great Britain & Ireland. Interim Report of Review Committee 1961.
4. Clarke, A. R. (1957). *Brit. Med. J.*, **2**, 722.
5. Clarke, A. R. (1959). *Ibid.* **1**, 125.
6. Moore, F. T. (1958). *Brit. J. Plast. Surg.*, **11**, 335.
7. Moynihan, F. J. (1961). *Brit. Med. J.*, **2**, 802.
8. Bourns, H. K. (1958). *Lancet*, **2**, 420.
9. Wesley-James, O. and Alder, V. G. (1961). *J. Clin. Path.*, **14**, 96.
10. Anderson, J. (1958). *Brit. Med. J.*, **2**, 1569.
11. Hewer, T. F. (1960). *Med. Journal of South West*, **75**, 14.
12. Graham Stack, H. (1960). *Brit. Med. J.*, **1**, 919.
13. Gairdner, D. (1949). *Ibid.*, **2**, 1433.
14. British Orthopaedic Association. Memorandum on Accident Services (1959).

